# Virtual Reality–based Methods for Training Novice Electrophysiology Trainees—A Pilot Study

**DOI:** 10.19102/icrm.2023.14091

**Published:** 2023-09-15

**Authors:** Benjamin Gorbaty, Susana Arango, David Buyck, Ryan C. James, Samantha T. Porter, Paul Iaizzo, Tjörvi E. Perry, Stephen Seslar

**Affiliations:** ^1^Division of Cardiothoracic Anesthesia, Department of Anesthesiology, University of Minnesota, Minneapolis, MN, USA; ^2^Department of Surgery, The Visible Heart^®^ Laboratories and the Institute for Engineering in Medicine, University of Minnesota, Minneapolis, MN, USA; ^3^Liberal Arts Technologies and Innovation Services, University of Minnesota, Minneapolis, MN, USA; ^4^Dopl Technologies, Seattle, WA, USA; ^5^Division of Pediatric Cardiology, University of Washington, Seattle Children’s Hospital, Seattle, WA, USA

**Keywords:** Anatomy, cardiac electrophysiology, medical education, virtual reality, 3-dimensional imaging

## Abstract

Developing an accurate and detailed 3-dimensional (3D) mental model of cardiac anatomy is critical for electrophysiology (EP) trainees. Due to its immersive nature, virtual reality (VR) may provide a better learning environment than traditional teaching methods for assimilating 3D cardiac anatomy. The purpose of this pilot study was to evaluate the technical feasibility of an interactive, remote VR-based method for teaching cardiac anatomy to novice EP trainees. We created a shared, remote VR environment that allows the shared viewing of high-resolution 3D cardiac models. Eighteen trainees accepted for pediatric and adult EP fellowships were recruited. We performed a cohort study comparing the traditional teaching methods with the VR learning environment. Participants completed a demographic questionnaire and a satisfaction survey. The adult EP trainees were given a multiple-choice pre- and post-test exam to assess their anatomical knowledge. Both the adult and pediatric EP trainee cohorts rated the VR experience positively and preferred the VR environment to the more traditional teaching method. All the participants expressed interest in incorporating the VR learning environment into the EP fellowship curriculum. The usability of the system was relatively low, with approximately one-third of participants rating the system as hard to use. The impact of the VR session on exam performance was mixed among the adult cohort. We demonstrated the feasibility of gathering geographically dispersed EP fellows in training with a shared VR-based environment to teach cardiac anatomy. Although we were not able to demonstrate a learning benefit over the traditional lecture format in the adult cohort, the training environment was favorably received by all the participants.

## Introduction

Developing an accurate and detailed 3-dimensional (3D) mental model of functional and relative cardiac anatomy is critical for new interventional electrophysiology (EP) trainees.^1^ The American College of Cardiology Foundation and the Heart Rhythm Society state that competence in invasive EP studies, catheter ablation, and cardioversion requires not only a wide spectrum of technical skills but also a thorough understanding of normal and pathological anatomy.^2,3^ Traditional teaching methods (classrooms, wet labs, textbooks, and lectures) may be suboptimal for this purpose^4^ and lack standardization and reproducibility across learners. These systems remain limited by the 2-dimensional display, which compromises understanding the complex 3D structure of the heart and the lack of detail, accuracy, and variability of the cardiac models.^5^ Due to its immersive, stereoscopic nature, virtual reality (VR) may provide a better learning environment for developing the spatial understanding of 3D cardiac anatomy necessary to perform EP procedures. The purpose of this pilot study was to test the technical feasibility of an interactive, remote VR-based method for teaching cardiac anatomy to novice EP trainees. Additionally, we compared the VR-based approach with a traditional lecture-based approach and attempted to gain insight on these experiences through formal user feedback.

## Methods

### Virtual cardiac models

For the adult EP trainee cohort, we visualized 3 different virtual human heart specimens generated as follows. In collaboration with the Visible Heart^®^ Laboratory and the Cardiothoracic and Vascular Education Center at the University of Minnesota (Minneapolis, MN, USA) and with appropriate consents from LifeSource, a local organ procurement organization (Minneapolis, MN, USA), we perfusion-fixed and gelled 3 explanted hearts in an end-diastolic state immediately following organ recovery.^6^ Micro-computed tomography (μCT) images (×3000) were obtained (North Star Imaging, Rogers, MN, USA) with a voxel resolution of 90–120 μm (which are resolutions that cannot be readily obtained during in vivo imaging).^7^ The anonymized datasets were imported into the Mimics^®^ Innovation Suite software (Materialise NV, Leuven, Belgium) for segmenting and generating a 3D computational model of the heart. Models were exported using the surface tessellation language (STL) format to Blender (Blender Foundation, Amsterdam, the Netherlands), for further processing. The poly-count was lowered for acceptable real-time use, imperfections were removed, and surfaces were smoothed to aid in visualizations.

For the pediatric EP trainee cohort, we sourced 2 additional human cardiac models as STL files supplied by Boston Children’s Hospital (Boston, MA, USA) and Mie University (Kii Peninsula, Japan). The first model was derived from a high-resolution CT scan of a cadaveric waxed-heart specimen with an unrepaired peri-membranous ventricular septal defect. The second was similarly obtained from a cadaveric waxed-heart specimen with an unrepaired common atrioventricular canal defect.

### The virtual reality learning environment

#### Virtual Heart Viewer

In collaboration with the Visible Heart^®^ Laboratory and the Advanced Imaging Service for Objects and Spaces at the University of Minnesota, using the game engine Unity (Unity Technologies, San Francisco, CA, USA), we developed a VR application for displaying the virtual heart models, as described previously. The models were imported as STL files, and a set of custom shaders were created to allow for dynamic cutting of cardiac anatomy in real time with a menu made available to switch between digitally generated predefined views. Using a VR headset and a pair of controllers (one per hand), users could “hold,” “rotate,” and “slice” the 3D heart and the surrounding vasculature along infinite planes, immediately gaining full access to the high-resolution surface and internal cardiac structure.

#### Shared virtual presence

**Figure 1:**
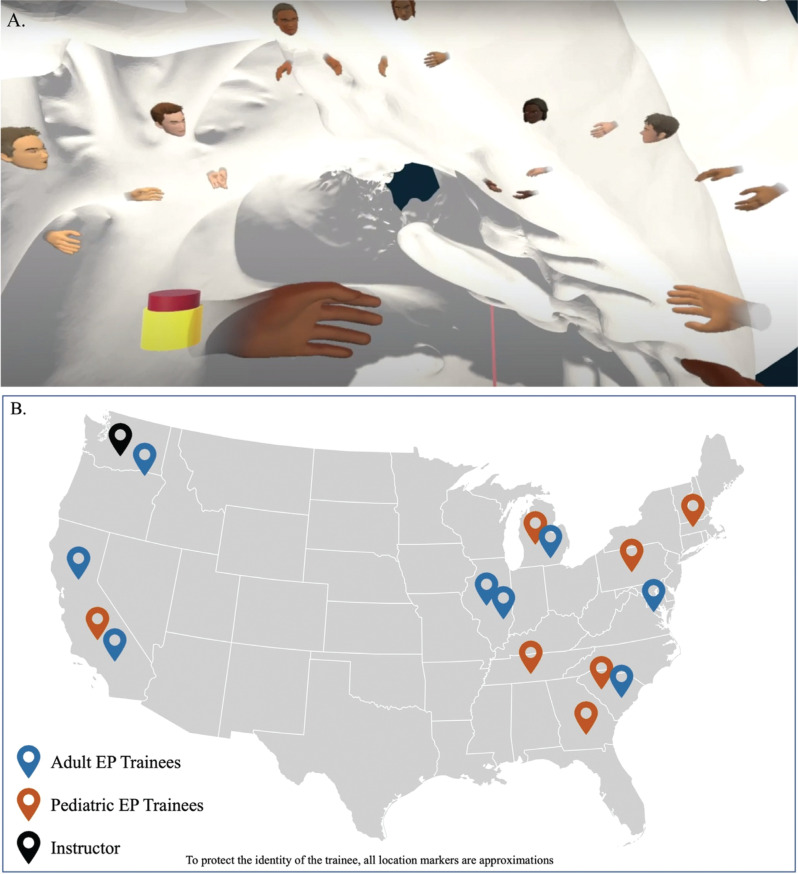
Participants and instructor gathered in the virtual training environment **(A)** from 15 training sites across the United States **(B)**. *Abbreviation:* EP, electrophysiology.

The Virtual Heart Viewer application described earlier was then integrated into Pluto™ VR, a commercially available shared virtual presence application, allowing multiple remote users to experience the Heart Viewer application simultaneously **(Figure 1, Video 1)**. The Pluto™ application renders users’ avatars capable of eye movement, facial expressions, and hand gestures and also allows the users to toggle between independent and synchronized views of the anatomy. All trainees and the instructor (S.P.S.) participated in the virtual learning environment using a Meta Quest or Quest2 head-mounted display (Meta, Menlo Park, CA, USA).

#### Cloud streaming

The Virtual Heart Viewer application and Pluto™ VR (Pluto VR Inc., Seattle, WA, USA) were run using PlutoSphere, a commercially available service that runs applications in a nearby data center and streams their rendered output to a VR headset. All trainees and the instructor (S.S.) had a dedicated PlutoSphere machine to run the necessary software and participated in the virtual learning environment using the PlutoSphere client application running on their Meta Quest or Quest2 head-mounted display.

### Participants

Novice adult and pediatric EP trainees were recruited from the U.S. Accreditation Council for Graduate Medical Education–accredited EP and Cardiology Fellowship Programs, respectively. Both adult and pediatric trainees had been accepted into the EP fellowship training but, with a single exception, had not yet started EP fellowship training. The study received approval from the University of Washington School of Medicine Institutional Review Board. Informed consent was obtained from each study participant.

### Study design

A pilot cohort study was performed to compare traditional teaching methods with the remote, shared VR learning environment. The adult EP trainees were given a multiple-choice pre-test covering right atrial anatomic relationships. They then received a 40-min conventional Microsoft PowerPoint (Microsoft Inc., Redmond, WA, USA) didactic lecture through an online video chat portal, followed by a post-test (post-test #1). On a separate day, within 1 week of the initial session, they participated in a 40-min training session covering the same content in the remote, shared virtual learning environment as described previously. Finally, within 48 h of participating in the virtual session, they completed a second post-test (post-test #2), a training satisfaction survey comparing conventional and VR-based learning methods, and a demographic questionnaire. The pre-test, post-test #1, and post-test #2 differed only in the order that the questions were presented.

**Figure 2:**
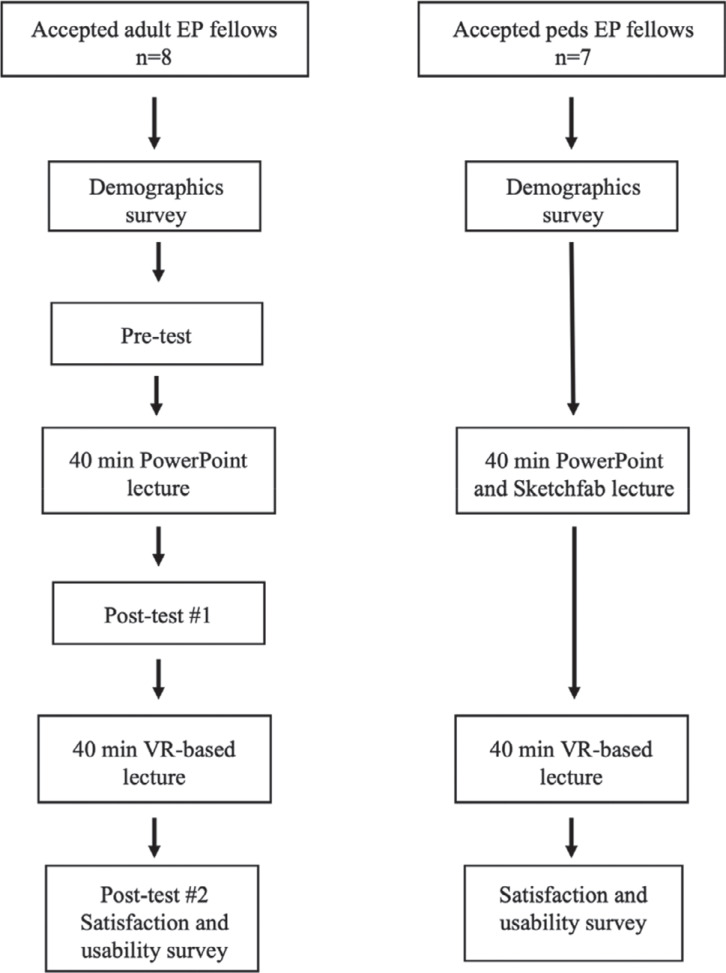
Study design and flow of participants through the study. *Abbreviations:* EP, electrophysiology; Peds, pediatric; VR, virtual reality.

Due to study time constraints, the pediatric EP trainees did not participate in the pre- or post-test. Like the adult EP trainees, they received a 40-min conventional Microsoft PowerPoint didactic lecture through an online video chat portal. Instead of the right atrial anatomy covered in the adult EP trainee group, the content of these lectures included the cardiac conduction system in normal hearts versus various types of ventricular septal defects. The 40-min didactic session was supplemented by an online 3D model viewing application (Sketchfab, Inc., New York, NY, USA) displaying the same congenital heart disease models viewed in the VR environment. In the same sitting, they next participated in a 40-min training session covering the same content in the remote, shared virtual learning environment, as described previously. Finally, within 48 h of participating in the virtual session, they completed the demographic and satisfaction surveys. Data were collected anonymously using online Google Forms (Google LLC, Mountain View, CA, USA) **(Figure 2)**.

### Data-collection instruments

#### Demographics form

Demographic data, including the subject’s age (years), sex (male or female), postgraduation year, current training position (specialty, pediatric vs. adult), prior experience in EP simulation training, clinical cardiac catheter ablation procedures, and prior VR experience (medical and recreational), were recorded **(Appendix 1)**.

#### Right atrial anatomy pre-test and post-test

The pre- and post-anatomy tests consisted of 20 multiple-choice questions designed to assess factual and inferential/relational understanding of normal right atrial cardiac anatomy relevant to clinical EP. The questions were adapted from those used in a previously published study.^8^ The only difference between the pre-test and post-test was the order in which the questions were presented. The participants did not receive feedback on their exam performance on any of the tests. Results were collected anonymously **(Appendix 2)**.

#### Training satisfaction survey

The participant satisfaction was assessed using a Likert survey adapted from the previously validated System Usability Scale^9^ and a validated measure of VR user experience **(Appendix 3)**.^10^

### Statistical analysis

Anonymized raw data were exported from Google Forms into Microsoft Excel for statistical analysis. Descriptive statistics were conducted. Continuous variables are expressed as mean ± standard deviation values.

## Results

### Study cohort

Ten adult and 8 pediatric EP trainees agreed to participate in the study. Three trainees were unable to complete the study because of logistical difficulties setting up their VR headsets in time for the study, leaving 8 adult and 7 pediatric EP trainees who completed all aspects of the study. By design, both the adult and pediatric groups consisted of novice trainees with limited experience performing EP procedures. In addition, most participants had little to no experience in VR **(Table 1)**.

### Pre- and post-test results (adult electrophysiology trainee cohort only)

**Figure 3:**
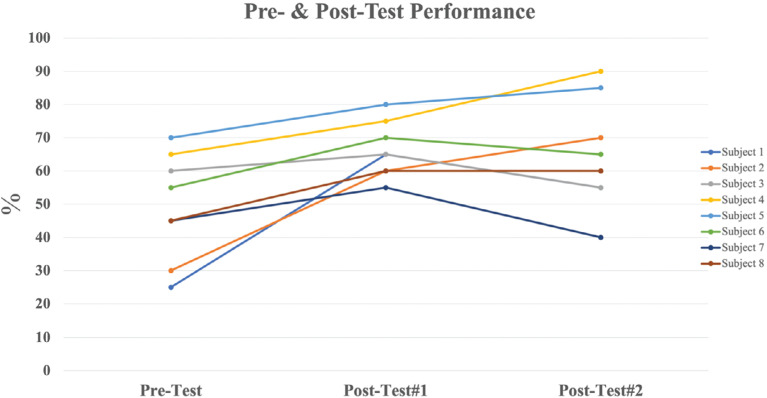
Pre- and post-test scores from the adult electrophysiology trainee cohort.

All participants improved their percentage of correct responses after the Microsoft PowerPoint presentation. The results after the VR experience were mixed; 3 participants improved their score, 1 participant achieved the same score, and 3 participants performed worse after the VR experience, respectively **(Figure 3)**.

### Participant satisfaction survey results

**Figure 4:**
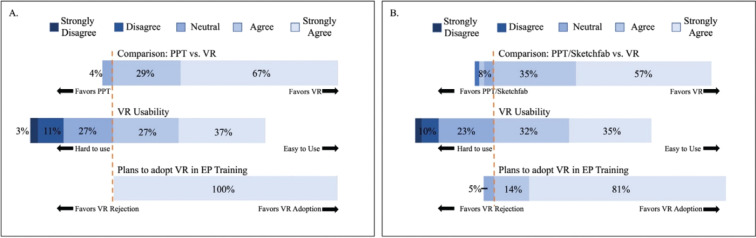
Aggregated Likert survey results from **(A)** adult EP trainees and **(B)** pediatric EP trainees. *Abbreviations:* EP, electrophysiology; PPT, Microsoft PowerPoint-based training session; Sketchfab, 3D model collections by heart models—Sketchfab; VR, virtual reality.

Overall, both the adult and pediatric EP trainee cohorts subjectively rated the VR experience positively and preferred the VR environment to the more traditional teaching methods. All the participants expressed an interest in incorporating the VR learning environment into the EP fellowship curriculum. The scores for the system’s usability were relatively lower **(Figure 4)**.

## Discussion

This study demonstrates the technical feasibility of bringing together a cohort of geographically dispersed adult and pediatric EP trainees (and instructors) into a shared, immersive 3D learning environment for viewing and interacting with high-resolution digital human heart models. In addition, this platform features high-resolution 3D digital models derived from human cadaveric specimens with and without structural congenital heart disease rather than artist-rendered digital models for unsurpassed accuracy and realism. Finally, this platform allowed every student to have an unimpeded view of the cardiac model from the perspective of the instructor as well as a shared avatar presence. While our results did not suggest a significant difference in knowledge acquisition when supplementing lecture-based learning with a VR-based experience, our results will allow us to appropriately power future randomized trials.

Studying cardiac anatomy in a 3D learning environment has been shown to improve spatial understanding.^11^ Similarly, the use of VR in learning cardiac anatomy significantly improved the performance of medical students on a post-test evaluation compared to that of control subjects who did not participate in the VR experience.^12^ Despite inadequate power, we anticipated an incremental improvement in post-test performance following exposure of the cohort to the shared VR learning environment; however, we were surprised to see that the test performance in most subjects either failed to improve or worsened after exposure to the VR environment. While the pilot nature of this study precludes a causal inference, it is worth speculating as to why this may have been so. Anecdotally, we noted that subjects whose post-test scores improved after exposure to the virtual learning environment generally had more hours of prior VR experience, raising the possibility that learning in a VR environment takes practice and exposure to VR. The lower usability scores seen in the satisfaction survey could have distracted students and impeded learning. For example, during 1 session, students were inadvertently grabbing the virtual heart model and repositioning it, requiring the instructor to repeatedly reposition it to continue the lesson.

More generally, usability was a significant issue reflected not only in the ratings but also in participation in the study. For example, 3 of 18 participants could not get their headsets to work and could not participate in the study. Of those who did participate, nearly one-third rated the VR system as hard to use. This indicates that the current generation of technology requires significant improvement before its widespread deployment. That being said, addressing the usability issues will be relatively straightforward from a technology development standpoint. In contrast, the path to measuring the subject’s learning from VR-based training is more challenging. It is not clear, for example, that the multiple-choice exam used in this study was the best means to assess improvements in spatial understanding of cardiac anatomy. Learning anatomy has been shown to enhance performance on standardized spatial ability tests.^13^ Consideration therefore might be given, for example, to designing spatial visualization tests specific to cardiac anatomy, potentially with them even being deployed in VR. In any case, for trainees in procedural fields such as EP, it will be critical to assess the value of the VR learning experience in the subsequent clinical procedural performance of trainees. While not presently available, a validated objective structured assessment of technical skills for EP trainees could be an important measure of the educational utility of the VR learning environment.

Finally, the importance of learner engagement in deriving value from an educational experience is well recognized.^14^ Educators are increasingly recognizing the value of designing medical education curricula to be enjoyable and even entertaining.^15,16^ Like Maresky et al.,^12^ we found high levels of user engagement in the virtual learning environment. For example, 78% of participants strongly agreed and the remainder agreed with the statement, “I felt stimulated by the virtual reality learning environment.” Further, 57% agreed or strongly agreed with the statement, “I became so involved in the virtual environment that I was not aware of things happening around me.”

### Limitations

This feasibility study was not designed to make statistically meaningful comparisons between different training methods due to its small sample size. In addition, we likely encountered selection bias because nearly all of our participants had little experience with VR technology. Similarly, the cohort design of the study in which the subjects repeated the same exam multiple times could have biased the results toward improved performance in subsequent exposures based on greater familiarity with the exam. In addition, due to the compressed study timeframe, the results of our analysis could have been impacted by learning fatigue due to repetitive exposure to similar content, anxiety regarding exposure to new technology, and repeated testing over a relatively short timespan. Finally, as discussed at length earlier, more work needs to be done to develop and validate tools to measure spatial learning and clinically meaningful outcomes of VR-based training.

### Next steps

Since this study was conducted, the VR learning environment has been integrated into the curriculum of 5 (4 adult and 1 pediatric) EP fellowship training programs in the United States for ongoing development and assessment. We will focus our efforts on refining the user experience and developing and validating meaningful learning assessment tools.

## Conclusion

This study demonstrates the feasibility of gathering geographically dispersed EP fellows in training in a shared, virtual, high-fidelity learning environment to immerse trainees in spatially complex cardiac anatomy. To demonstrate the value of a VR-based educational platform, we will need to continue to refine the user experience, facilitate greater exposure to a VR environment, and then develop and validate meaningful assessment tools.

## Figures and Tables

**Video 1:** Virtual reality teaching session.

**Table 1:** Demographic Survey Results

Subject Characteristics	Adult EP Trainees (n = 8)	Pediatric EP Trainees (n = 7)
Age (years)	35 (4.5)	33
Female sex	25%	25%
PGY (years)	6 (0.5)	6 (0.7)
Current position		
Cardiology fellow (second year)	0	2
Cardiology fellow (third year)	7	4
Cardiology fellow (fourth year)	1	0
EP fellow (first year)	0	*1*
Mapping: simulation (no. of hours in the past year)		
None	3	4
<3	5	3
Mapping: clinical (no. of procedures in past year)		
None	0	1
<3	7	2
3–25	0	2
26–50	1	0
>50	0	2
VR in health care (no. of hours in past year)		
None	8	5
<3	0	1
3–25	0	1
VR in recreation (no. of hours in past year)		
None	6	4
<3	1	0
3–25	0	3
26–50	1	0

*Abbreviations:* EP, electrophysiology; no., number; PGY, postgraduation year; VR, virtual reality.

**Image 5.**
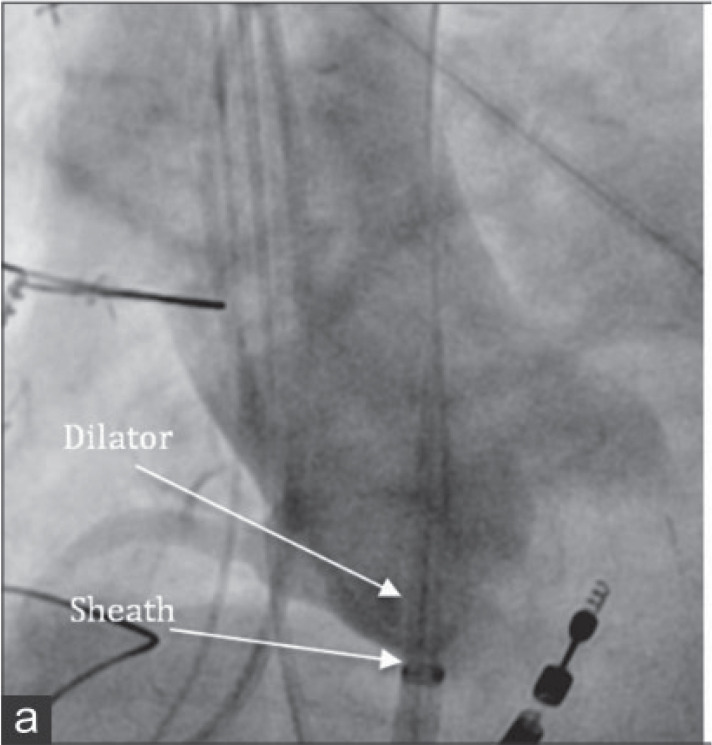
Republished with permission from Cao Y, Rosenthal E, Qureshi SA. Inadvertent ascending aortic perforation after transseptal puncture: Successful treatment with an Amplatzer Duct Occluder II device. *Ann Pediatr Cardiol*. 2019;12(2):138–140. https://journals.lww.com/aopc/fulltext/2019/12020/inadvertent_ascending_aortic_perforation_after.12.aspx.

## Appendix 1. Virtual Reality in Electrophysiology Training Pilot Study Demographics Form

Name: _____ (Recorded only to confirm completion. Your name will not be recorded with the data you submit on this form.)

1. Age: _____ years
2. Sex: _____Female  _____ Male
3. PGY _____ 1 _____ 2 _____ 3 _____ 4 _____ 5 _____ 6 _____ 7 _____ 8 _____ 9 _____ 10 _____>10
4. Current Position
  **Table:**
  Adult-trained	Pediatric-trained
  _____Cardiology Fellow (1st year)	_____Cardiology Fellow (1st year)
  _____Cardiology Fellow (2nd year)	_____Cardiology Fellow (2nd year)
  _____Cardiology Fellow (3rd year)	_____Cardiology Fellow (3rd year)
  _____Cardiology Fellow (4th year)	_____Cardiology Fellow (4th year)
  _____EP Fellow (1st year)	_____EP Fellow (1st year)
  _____EP Fellow (2st year)	_____EP Fellow (2st year)
  _____Other	_____Other
5. Prior experience in EP mapping (catheter manipulation) during *simulation training* (# of hours of simulated catheter movement practice in the past year):
  _____ None _____ <3 ____ 3–25 _____ 26–50 _____ >50   hours
6. Prior experience in EP mapping (catheter manipulation) *during clinical catheter ablation procedures* (# of procedures in the past year):
  _____ None _____<3 _____3–25 _____25–50 _____>50   procedures
7. Prior Virtual Reality Experience in healthcare simulation (# of hours in the past year):
  _____None _____<3 _____3–25 _____25–50 _____>50   hours
8. Prior Virtual Reality Experience in recreational activities (# of hours in the past year):
  _____None _____<3 _____3–25 _____25–50 _____>50   hours
9. (OPTIONAL) List at least one additional feature you would like to see added to the VR learning environment you participated in as part of this study:
  _____________________________________________________________________________
  _____________________________________________________________________________
  _____________________________________________________________________________
  _____________________________________________________________________________

## Appendix 2. Virtual Reality in Electrophysiology Training Pilot Study (Part I: Pre-test)

Answer questions 1–3 using Image 1:

1. At what structure is the RED arrow pointing? (Knowledge)
  - Aorta
  - SVC
  - Right upper pulmonary vein
  - RAA
  - IVC
2. Which structure would be furthest away from a TEE probe positioned behind the left atrium (not shown) in the anterior–posterior dimension? (Comprehension)
  - Coronary sinus
  - Tricuspid valve annulus
  - Right atrial appendage
  - Inferior vena cava
  - Crista terminalis
3. What information about the catheter position is NOT conveyed by the image? (Comprehension)
  - Superior/inferior position
  - Septal/lateral position
  - Anterior/posterior
  - None of the above
4. A patient has an ectopic atrial focus firing from the right atrial appendage (RAA). Relative to the sinus rhythm P-wave polarity in V1, the initial forces will be directed: (Comprehension)
  - More posteriorly because the RAA is predominantly anterior to the sinus node
  - More inferiorly because the RAA is predominantly superior to the sinus node
  - No different than the sinus node in lead V1
  - More leftward because the RAA is predominantly rightward of the sinus node
  Answer questions 5 and 6 using Image 2:
5. What would you expect to see in the ultrasound image based on the ICE fan indicated by the red arrow? (Comprehension)
  - The smooth endocardial surface of the sinus venosus
  - The trabeculated crista terminalis and right atrial appendage
  - The lateral cavo-tricuspid isthmus
  - The right upper pulmonary veins
6. An ablation in the roof of the RA (purple arrow) successfully results in elimination of an atrial tachycardia but also leads to sinus bradycardia with sinus pauses that recovers over the next 48 hours. What is the most likely mechanism of SA node dysfunction?
  - Direct injury to the SA node
  - Injury to the parasympathetic ganglion
  - Injury to the SA nodal artery
  - Unmasked underlying sinus node dysfunction following elimination of the atrial tachycardia
7. The thickened ridge at the superior margin of the fossa ovalis is: (Knowledge)
  - Septum primum
  - Part of the interatrial septum
  - Septum secundum
  - Part of the Thebesian valve
  Answer questions 8–10 using Image 3:
8. A patient presents for typical cavo-tricuspid isthmus (CTI) dependent flutter catheter ablation. You note on ICE that this patient has prominent pectinate muscles extending from the crista terminalis into the region of the CTI. Based on this, you should place your CTI line: (Comprehension)
  - On the medial side of the CTI (where catheter is currently shown)
  - On the lateral side because the CTI will be less trabeculated there
  - Using an 8-mm-tip ablation catheter to make a deeper lesion
  - Starting just anterior to the eustachian ridge
9. Relative to the position of the tip of the catheter, the coronary sinus is: (Comprehension)
  - Anterior and medial
  - Posterior and inferior
  - Rightward and superior
  - Posterior and medial
10. While performing a pull-back ablation line along the medial CTI line, the catheter tip moves superiorly, and tip contact force diminishes. You suspect that this happens because:
  - There are prominent pectinate muscles here
  - There is a prominent Thebesian valve
  - There is a prominent eustachian valve
  - The catheter tip is trapped in a deep eustachian pouch
  Answer questions 11–13 using Image 4:
11. What is the primary function of the blue structure in the drawing? (Knowledge)
  - Atrio-ventricular conduction
  - Intra-atrial conduction
  - Inter-atrial conduction
  - Arterial blood supply to the sinus node
12. What structure lies anterior–inferior to the orange arrow at the level of the superior TV annulus? (Comprehension)
  - SA node
  - Fossa ovalis
  - Coronary sinus
  - Bundle of His
13. During a retrograde approach to the LV, the ablation tip records an atrial electrogram. Where is the catheter tip most likely positioned during this recording? (Comprehension)
  - Retrograde through the aortic and mitral valves into the left atrium
  - Non-coronary cusp
  - Right coronary artery
  - Area of aortic-mitral valve continuity
  Answer questions 14 and 15 using Image 5:
14. An arterial pressure wave is seen, and angiography is shown above following transseptal puncture. The most likely error during the puncture was that the transseptal needle was oriented too: (Comprehension)
  - Posteriorly
  - Inferiorly
  - Medially
  - Anteriorly
15. Which aortic root cusp was likely punctured? (Comprehension)
  - NCC
  - LCC
  - RCC
  - None of the above—the transseptal sheath is in the pulmonary root
  Answer questions 16 and 17 using Image 6:
16. What chamber/vessel might your catheter tip enter if you applied force at the red arrows? (Comprehension)
  - LA
  - Pericardial space
  - RV
  - CS
  - Aorta
17. What structure is near the tip of the BLUE arrow? (Knowledge)
  - Eustachian ridge
  - IVC
  - Thebesian valve
  - CTI
  - Vieussens valve
  Answer question 18 using Image 7:
18. An ectopic atrial tachycardia maps to the posterior SVC/RA junction (orange arrow), but ablation at this location is ineffective. What should the operator do next? (Comprehension)
  - Switch to an irrigated-tip ablation catheter to create a deeper lesion
  - Perform transseptal puncture
  - Try approaching with the ablation catheter coming from the RIJ
  - Perform a cavo-tricuspid isthmus (CTI) ablation line
  Answer questions 19 and 20 using Image 8:
19. An ICE catheter in the right atrium creates the image above. What structure is seen at the orange arrow? (Knowledge)
  - Pulmonary artery
  - Esophagus
  - Aortic root
  - Descending aorta
20. What would you tell the operator that is about to perform a transseptal puncture at this location (see white arrow? (Comprehension)
  - The needle is directed too posteriorly
  - The needle is directed too anteriorly
  - The needle is positioned in an optimal location
  - The needle is positioned too superiorly

## Appendix

**Appendix 3.** Virtual Reality in Electrophysiology Training Pilot Study Satisfaction Survey

Question Type	Questions	Strongly Disagree	Disagree	Neutral	Agree	Strongly Agree
		1	2	3	4	5
Usability	1. My interactions with the virtual reality learning environment seemed natural.					
Comparative	2. The virtual reality learning environment added to my 3D spatial awareness of cardiac anatomy beyond what I took away from the PPT lecture*.*					
Usability	3. I found the virtual reality learning environment unnecessarily complex.					
Usability	4. I think that I would like to use the virtual reality learning environment frequently.					
Usability	5. Controlling objects in the virtual reality learning environment seemed natural.					
Comparative	6. Compared with virtual reality, PPT lectures are a better way to learn cardiac anatomy.					
Usability	7. I thought the virtual reality learning environment was easy to use.					
VR Environment	8. Interacting with the other avatars in the virtual reality learning environment made the experience more enjoyable to me.					
Comparative	9. I preferred the virtual reality learning experience to the PPT session.					
Usability	10. I think that I would need the support of a technical person to be able to use virtual reality learning environment.					
Study Design	11. Both the virtual reality and PPT sessions were too basic for me, and I did not gain much from either session.					
Usability	12. I found the virtual reality learning environment very cumbersome to use.					
Usability	13. I experienced symptoms of dizziness, blurry vision, nausea, or other unpleasant sensations that would make me reluctant to use virtual reality for future learning sessions.					
Study Design	14. I feel that the pilot study’s pre- and post-tests were effective assessment tools to measure what I took away from this learning experience.					
Application	15. I would like to see additional virtual reality EP content developed, such as anatomic variants, congenital heart disease specimens, or models with electrical signal propagation.					
Comparative	16. PPT-style lectures and the virtual reality learning experiences are synergistic.					
Usability	17. I thought there was too much inconsistency in this virtual reality learning environment.					
Application	18. If available to me, I would use the virtual reality learning environment for self-directed study.					
Usability	19. I would imagine that most people would learn to use the virtual reality learning environment very quickly.					
Usability	20. I became so involved in the virtual environment that I was not aware of things happening around me.					
Usability	21. I found that the various functions in this virtual reality learning environment were well integrated.					
Usability	22. Getting into the virtual reality learning experience was cumbersome and time-consuming.					
Application	23. Personally, I would say the virtual environment is impractical for learning.					
Usability	24. I felt stimulated by the virtual reality learning environment.					
Usability	25. I felt very confident using the virtual reality learning environment.					
Application	26. I would like to see virtual reality-based learning experiences incorporated into my EP Fellowship’s core didactic lecture series.					
Usability	27. I would need to learn a lot of things before I could get going with this virtual reality learning environment.					
VR Environment	28. I found the virtual environment amateurish.					
Usability	29. Learning to operate the virtual environment would be easy for me.					
VR Environment	30. Virtual reality in its present state of development is better suited for video games and is not yet appropriate for electrophysiology training.					

(OPTIONAL) List at least one additional feature you would like to see added to the VR learning environment you experienced as part of this study:

_______________________________________________________________________________________

_______________________________________________________________________________________

(OPTIONAL) Any overall feedback regarding the study?

_______________________________________________________________________________________

_______________________________________________________________________________________
